# Tetracycline and Oxacillin Act Synergistically on Biofilms and Display Increased Efficacy In Vivo Against *Staphylococcus aureus*

**DOI:** 10.1007/s00284-024-03959-4

**Published:** 2024-11-06

**Authors:** Amy K. Tooke, Rebecca E. Hodges, Josie F. Pyrah, Kenneth W. Bayles, Stephen A. Renshaw, Simon J. Foster

**Affiliations:** 1 School of Biosciences, University of Sheffield, Sheffield, S10 2TH UK; 2 Microbes in Health and Disease Theme, Newcastle University Biosciences Institute, Newcastle University, Newcastle upon Tyne, NE2 4HH UK; 3 Department of Pathology and Microbiology, University of Nebraska Medical Center, Omaha, NE USA; 4 The Bateson Centre, University of Sheffield, Western Bank, Sheffield, S10 2TH UK; 5 School of Medicine and Population Health, University of Sheffield, Beech Hill Road, Sheffield, S10 2RX UK; 6 Florey Institute, University of Sheffield, Sheffield, S10 2TH UK

## Abstract

Oxacillin (bactericidal) and tetracycline (bacteriostatic) are clinically relevant antibiotics that are routinely prescribed to treat *Staphylococcus aureus* infections but not conventionally used in combination. There is an urgent need for treatment regimens that can act upon biofilms during infection, associated with chronic infections on indwelling devices, as well as acute planktonic (systemic) infection. Here we show that in an in vitro model oxacillin and tetracycline act synergistically against *S. aureus* UAMS-1 biofilms, reducing the concentration of both antibiotics necessary to eradicate an established biofilm. Using an in vivo zebrafish larval infection model with *S.* *aureus* NewHG, they display improved bacterial clearance compared to each drug alone and can counteract a loss of host phagocytes, an important innate defence against *S. aureus*. In these cases, the bacteriostatic nature of tetracycline enhances rather than dampens the bactericidal action of oxacillin, although an exact mechanism remains to be elucidated. We suggest a dual therapy could be of clinical use against biofilm-forming *S. aureus* and has a potential use in patients with a compromised immune system.

**Supplementary Information:**

The online version contains supplementary material available at 10.1007/s00284-024-03959-4.

## Introduction

There is an urgent need to develop new antimicrobials given the global burden of antimicrobial resistance with *Staphylococcus aureus*, specifically methicillin-resistant *S. aureus* (MRSA) infections, responsible for 100,000 deaths worldwide in 2019 [1], but given that not all *S. aureus* infections are caused by MRSA, we can also refine and adapt existing treatments to prolong the usefulness of clinically approved drugs before resistance develops in all strains. Combining existing antibiotics is one such approach, which can have synergistic or undesirably, antagonistic effects, or have a neutral additive relationship [2]. Adjuvants can be used to amplify the efficacy of drugs including antibiotics [3, 4], but we can also use two or more antibiotics with different mechanisms to achieve similar outcomes. Additionally, the use of multiple drugs reduces the likelihood of resistance developing [2]. We sought to utilise two different experimental models to explore whether tetracycline and oxacillin, which are not conventionally combined, would act synergistically in combination against MSSA strains across two different contexts: an in vitro biofilm model, and an in vivo zebrafish infection model. These drugs showed efficacy individually in the zebrafish model, where they are both non-toxic (at the concentrations used) and able to penetrate within the embryos [5]. As these two classes of antibiotic are amongst the most commonly prescribed in England [6–8], their combination could have potentially useful applications. Tetracyclines are broad spectrum bacteriostatic protein synthesis inhibitors in clinical use since the 1950s, and oxacillin is a bactericidal beta-lactam (β-lactam)/penicillin that is routinely prescribed to treat methicillin-sensitive *S. aureus* (MSSA) infections. Tetracycline resistance is mainly through efflux, whereas β-lactam resistance can be mediated through modification of the penicillin binding proteins (PBPs) that the drugs target, and bacteria can resist both classes of antibiotics by producing degradative enzymes to inactivate the drugs [9]. It is thought that combining bacteriostatic (such as tetracycline) with bactericidal drugs would be counterintuitive as bactericidal agents tend to target actively growing cells resulting in antagonism [10], but there is current interest in combining tetracyclines with bactericidal antibiotics, such as omadacycline with rifampicin, which displays synergy and avoids development of rifampicin resistance in staphylococcal biofilms [11]. Therefore, as oxacillin is bactericidal, it is a good candidate to investigate alongside tetracycline.

Biofilm formation by *S. aureus* is a literal barrier to successful antibiotic treatment, with a key characteristic of biofilms being recalcitrance, whereby concentrations of antibiotics that would be effective against planktonic cells of the same genotype do not affect the same cells in a biofilm; furthermore, this effect is compounded as the biofilm grows and matures [12–15]. *S. aureus* biofilms are associated with chronic infection when they can form on medical implants as well as host tissues (such as during osteomyelitis, chronic wounds and infective endocarditis) and can act as bacterial reservoirs [16]. Given the fact that the ability to perform the phenotypic switch from planktonic to biofilm growth (and back again) is a key virulence mechanism that allows *S. aureus* to cause a variety of diseases, it is therefore of clinical importance to develop treatments that can eradicate biofilm infections, not just planktonic cells during systemic acute infections [16–19]. Bacterial cells released from biofilm are often sensitive to antibiotics that would not have affected them whilst in the biofilm; therefore, clearance and dispersal of biofilm before antibiotic treatment is a potential approach to cure biofilm-related infections [15].

Here we investigate how tetracycline and oxacillin behave against biofilms individually and in combination, demonstrating a synergistic relationship between the two drugs in an in vitro microfluidics system that act to eradicate established biofilms. Additionally, we use a larval zebrafish infection model, allowing insight into the host–pathogen interactions as larvae develop an innate immune system with similarity to mammalian systems [20–22]. We utilised the model with antibiotics known to diffuse into zebrafish therefore avoiding potential limitations whereby some drugs are unable to diffuse into larvae or are otherwise toxic [5, 23]. We show that the combination of oxacillin with tetracycline increased efficacy in the zebrafish model to control bacterial load in conjunction with the innate immune system. Finally, we demonstrate that the combination of tetracycline with oxacillin can rescue *S. aureus-*infected zebrafish larvae that have knocked down development of phagocytes, indicating that the antibiotics in this instance do more to control infection than cellular immunity.

## Materials and Methods

### Bacterial Strains and Growth Conditions used in this Study

We used two different strains of *S. aureus* in this study depending on the growth context. NewHG (SJF 3663) (Newman with *saeS*^*L*^ allele from strain RN1 [24]) is well characterised and was used for the zebrafish infection studies. The laboratory MSSA strain Newman has a mutation resulting in a constitutively active *saeS*^*P*^ which leads to increased virulence due to activation of certain virulence-related genes [24]. Replacement of the mutated allele to give strain NewHG restored parental virulence determinant levels.

UAMS-1 is a clinical osteomyelitis isolate that is well characterised for its biofilm growth [25].

Both strains NewHG and UAMS-1 are sensitive to oxacillin and tetracycline. Unless otherwise stated, *S. aureus* was grown at 37°C in tryptone soy broth (TSB, Oxoid), made using 30 g/l in deionised water and sterilised by autoclaving. Bacteriological agar (VWR) was added at 1.5 % w/v to TSB to make tryptone soy agar (TSA).

### Plasmids used in this Study

pCM29 [26] was transformed into UAMS-1 for constitutive cytosolic GFP expression in order to quantify area coverage in the Bioflux experiments.

### In vitro Biofilm Growth and Imaging

The model for dynamic *S. aureus* biofilm, as previously described [27], was used for the assessment of biofilm treatment by tetracycline and oxacillin. The Bioflux 1000 (Fluxion biosciences Inc.) system and Bioflux 1000 48-well plates (Fluxion Biosciences Inc.) and sterile 50% TSB were used for all experiments. 250-ml conical flasks containing 25 ml sterile TSB were inoculated from overnight cultures of UAMS-1 pCM29 to OD_600_ 0.05 (approximately 1x10^7^ CFU/ml) and incubated at 37°C, with shaking at 250 rpm until an OD_600_ 0.8 was reached. Biofilm growth channels were primed by adding 200 µl media to output wells and using reverse flow for 2 minutes at 10.0 dynes cm^2^. A 300 µl volume of fresh media was added to input wells and media in output wells replaced with *S. aureus* inoculum. The growth channels were then seeded by applying reverse flow of 2.0 dynes cm^2^ for ~2 seconds using guidance via microscope. The seeded plate was incubated for 1 hour at 37° C on the heated stage of the Bioflux 1000 system to allow attachment of *S. aureus* cells to the growth channels. 1 ml of fresh media was added to input wells and the remaining inoculum was aspirated from the output wells. Forward flow of 0.6 dynes cm^2^ was applied to all channels in the plate for 12 hours. After 12-h flow of media was paused, the manifold was removed from plate in situ and 1 ml of media added to input wells containing 1.2 x concentration of antibiotic to give a final volume of 1.2 ml of media containing a final concentration of antibiotic as detailed. Manifold was replaced and forward flow was resumed at 0.6 dynes cm^2^ for 12 hours. Bright-field and epifluorescence images were taken at 5-minute intervals for a total of 289 timepoints. All epifluorescence images monitoring GFP fluorescence were taken with a fluorescein isothiocyanate (FITC) filter. All biofilm treatments were repeated for a minimum of three independent experiments containing at least two technical replicates as has been previously established [28].

### Quantification of Acquired Biofilm Images

Images were reviewed using Bioflux Montage software (Fluxion Biosciences Inc.). Bright-field and epifluorescence images were calibrated to 0.32 µm/pixel. The open-source image processing software Fiji [29] was used for the quantification of area coverage. Area coverage was calculated from cytosolic GFP expression, using a macro to apply the same conditions to all timepoints for every field of view acquired across all experiments. Background signal was removed by thresholding the image to a minimum of 200 AU and all pixels between 200 and 63270 were included in the area measurement.

### Growth Conditions of *S. aureus* for Zebrafish Infection

Frozen stocks of *S. aureus* were streaked out onto TSA plates and incubated overnight at 37°C. A single colony was picked from the plate and inoculated into 10 ml TSB and incubated overnight at 37°C, with shaking at 250 rpm. The culture was diluted the following day to OD_600_ 0.05 in 50 ml TSB and grown at 37°C, with shaking at 250 rpm until it reached OD_600_ 1 after approximately 2 hours. The culture was prepared for injection into zebrafish as detailed in [20].

### In vivo Zebrafish Infection Experiments

Animal work was carried out according to guidelines and legislation set out in UK law in the Animals (Scientific Procedures) Act 1986, under Project License PPL 40/3574. Larvae were infected with 1500 CFU NewHG *S. aureus* at 33 hours post fertilisation (hpf) as previously described in [20] and survival monitored until 92 hours post infection (hpi), at which point larvae were culled. Larvae were maintained in E3 buffer prepared from a 10x stock solution diluted in deionised water, resulting in a final concentration of 5 mM NaCl, 0.17 mM KCl, 0.33 mM CaCl_2_, 0.33 mM MgSO_4_ with methylene blue added as an antifungal agent to a final concentration of 0.00005% w/v. The solution was autoclaved to sterilise and cooled to 28.5°C before use. Bacterial burden was determined by homogenising a sample of living larvae and all dead larvae in 100 µl E3 in sterile homogenisation tubes (AlphaLaboratories) containing sterilised 1.4 mm ceramic beads (Peqlab). Larvae were frozen or kept on ice to cool before being homogenised for 20 seconds via a FastPrep-24 homogeniser (MP Biomedicals). 20 µl homogenate was added to 180 µl PBS and serially diluted before being plated out as 10 µl spots onto tryptone soy agar (TSA) plates. Plates were incubated at 37°C overnight. The mean number of CFUs per 10 µl spot was determined to calculate CFU per larva.

### Treatment with Antibiotics

Larvae treated at 1 day post infection (dpi) were put into 96-well plates with 250 µl E3 per well, and then 50 µl E3 containing the antibiotic at 6x concentration was pipetted into the well. Larvae treated at 0 hpi in the morpholino experiment were immersed in E3 containing the antibiotics at the required concentrations and then dispersed into 96-well plates.

### Zebrafish Morpholino Experiments

*Tg(BACmpx:gfp)i114* zebrafish embryos at the 1-cell stage were injected with either 0.5 pmole (in 0.5 nl) pu.1 morpholino (ZDB-MRPHLNO-050224-1) or standard control morpholino (GeneTools) (whereby phagocytes develop normally). Morphants were infected with a target dose of 1500 CFU *S. aureus* NewHG at 33 hpf and left untreated as a control, or treated with 50 µg ml^-1^ tetracycline and 32 µg ml^-1^ oxacillin at 1 hpi. The following day at 18 hpi, survival was calculated for each group and morphants were homogenised to determine the bacterial load. Treatment was given early, as without phagocytes (as in the untreated group), the infection quickly overwhelms the embryos. It has been previously shown that embryos injected with pu.1 morpholino but then uninfected have no survival defect [22].

### Statistical Analysis

Statistics were carried out using GraphPad Prism (versions 9 and 10). Biofilm area coverage comparisons were made by Mann–Whitney test for the final timepoints (10.5 h). Zebrafish survival is represented using Kaplan–Meier survival curves, with comparisons between curves performed using the Log-Rank (Mantel-Cox) test. For a reduction in mortality from 40 % to 80 % survival, required sample size is 20 per group (confidence 80%, significance 5%, Chi squared). For the morpholino data to detect a reduction in proportions expected from 90% to 60%, 29 are required in each group (confidence 80%, significance 0.05, Chi squared).

## Results and Discussion

**Fig. 1:**
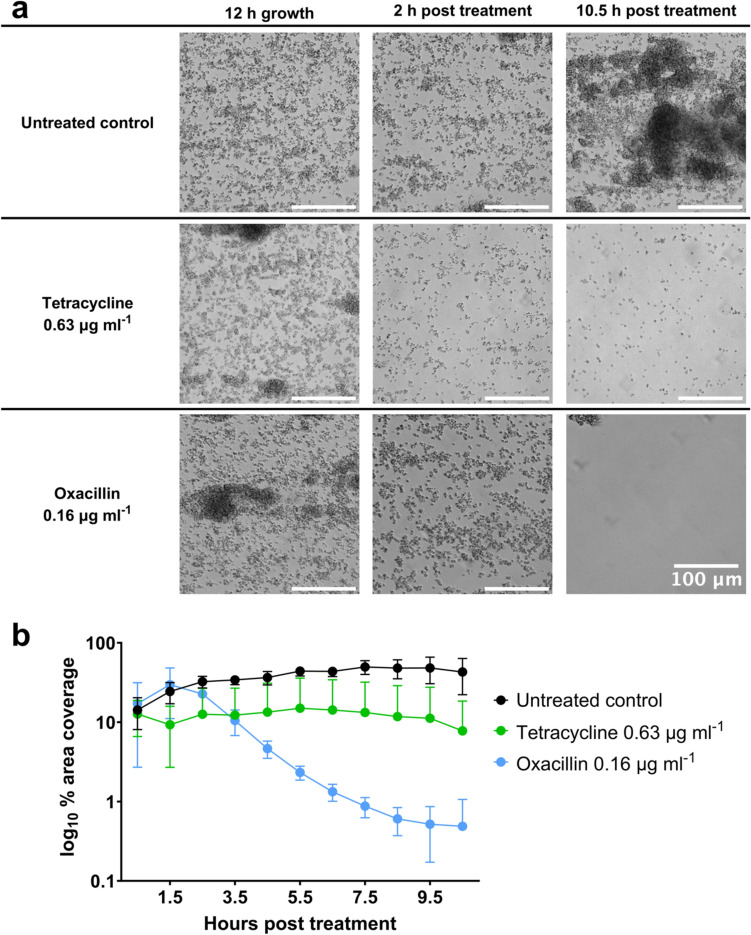
Effects of treating *S. aureus* biofilm with inhibitory (“high”) concentrations of tetracycline and oxacillin**. a** WT *S. aureus* strain UAMS-1 containing the pCM29 plasmid for constitutive cytosolic GFP expression was grown in the Bioflux system where bright-field and epifluorescence images were acquired at 5-minute intervals at 200 x magnification. Images shown were taken at 12 hours of biofilm development prior to treatment, 2 hours post treatment and 10.5 hours post treatment with tetracycline 0.63 µg ml^-1^, or oxacillin 0.16 µg ml^-1^. The scale bar in all images represents 100 µm. **b** Graph depicts quantification of biofilm area via measurement of cytosolic GFP from epifluorescence images taken at 5-minute intervals. The data represent the mean from 3 independent experiments containing at least 2 technical replicates, and error bars represent standard deviation. Reduction in biofilm formation for each treatment and the untreated control was *P* < 0.0001 for both antibiotics at 10.5 hours

**Fig. 2:**
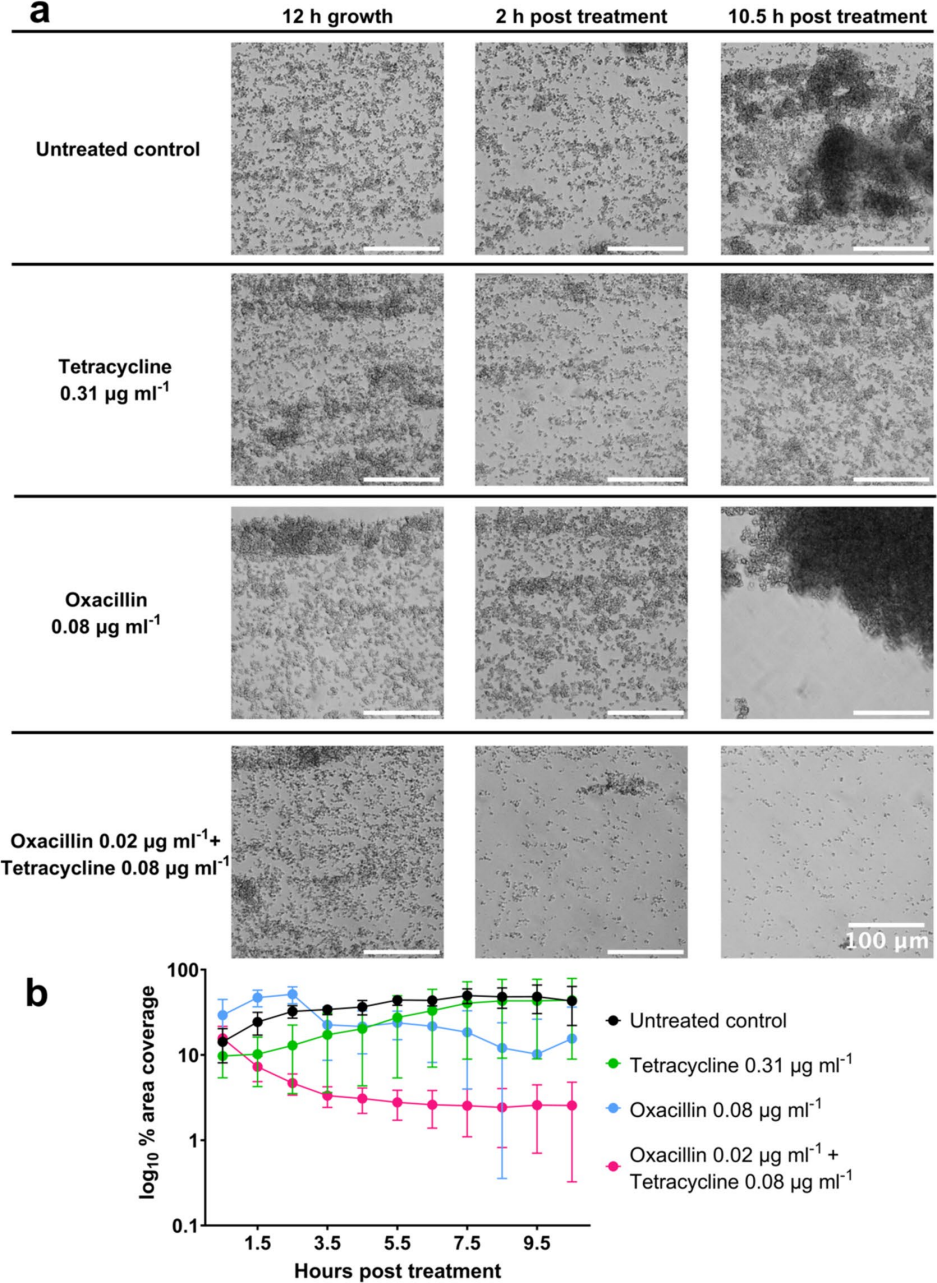
Effects of treating an *S. aureus* biofilm with oxacillin and/or tetracycline**.** WT *S. aureus* strain UAMS-1 containing the pCM29 plasmid for constitutive cytosolic GFP expression was grown in the Bioflux system where bright-field and epifluorescence images were acquired at 5-min intervals at 200 x magnification. **a** Images shown were taken at 12 hours of biofilm development prior to treatment, 2 hours post treatment and 10.5 hours post treatment with tetracycline 0.31 µg ml^-1^, oxacillin 0.08 µg ml^-1^ or tetracycline 0.08 µg ml^-1^ + oxacillin 0.02 µg ml^-1^, compared to an untreated control (top). The scale bar in all images represents 100 µm. **b** Graph depicts quantification of biofilm area via measurement of cytosolic GFP from epifluorescence images taken at 5-minute intervals. Concentrations in the combined group are 4 times lower than sub-inhibitory individual values displayed for tetracycline and oxacillin. The data represent the mean from 3 independent experiments containing at least 2 technical replicates, and error bars represent standard deviation. Reduction in biofilm formation between each single treatment and the combination was  *p* = 0.0653 for tetracycline and *p* = 0.2523 for oxacillin at 10.5 hours

In vitro methods were used to investigate whether tetracycline and oxacillin display any interactions when combined in vitro, which indicated a trend towards a synergistic effect (Supplementary Figure S.1). An E-test (bioMerieux) synergy assay was carried out whereby antibiotics at defined concentrations in the strip diffuse into the surrounding agar. This resulted in the characteristic pattern of growth inhibition when 2 strips are combined at 90° at the original MICs, with MIC of tetracycline decreasing from 1 μg ml^-1^ to 0.25-0.5 μg ml^-1^ in combination, and likewise oxacillin 0.19 μg ml^-1^ to 0.125 μg ml^-1^ (Supplementary Figure S.1 a). Similarly, a disk diffusion synergy assay resulted in a pattern of growth inhibition when tetracycline was combined with oxacillin indicative of synergy (Supplementary Figure S.1 b). Finally, a microplate checkerboard assay was carried out, resulting in a fractional inhibitory concentration (FIC) of 0.5625; a synergistic relationship is defined as FIC < 0.5 [30, 31]. To investigate this further, the BioFlux 48-well system was used to visualise the effects of these treatments individually and in combination against *S. aureus* biofilms. UAMS-1 *S. aureus* (MSSA) biofilms were established over 12 hours prior to treatment with tetracycline at 0.63 (high) or 0.31 (mid) µg ml^-1^ or oxacillin at 0.16 (high) or 0.08 (mid) µg ml^-1^ and subsequently cultured for a further 12 hours. Treatment with high doses of tetracycline was bacteriostatic and oxacillin was able to eradicate biofilms, whereas when left untreated the biofilms continue to develop and increase in visible mass and coverage of the field of view (Figure 1). Mann–Whitney test comparisons between each treatment and the untreated control were p<0.0001 for both antibiotics at 10.5 hours. Treatment with mid-concentrations of tetracycline or oxacillin was sub-inhibitory (Figure 2). Low concentrations (0.25x concentration of mid-doses) of tetracycline (0.08 µg ml^-1^) and oxacillin (0.02 µg ml^-1^) that were individually sub-inhibitory (data not shown) were used in combination and were able to eradicate the biofilms (Figure 2). Mann–Whitney test comparisons between each treatment and the combination were *P* = 0.0653 for tetracycline and *P* = 0.2523 for oxacillin at 10.5 hours. Taking the minimum inhibitory concentration (MIC) as between the high and mid-doses results in a FIC in the range of 0.25–0.5, indicating synergy. These results demonstrate a clear synergistic, rather than additive, relationship between the two antibiotics in this context.

**Fig. 3:**
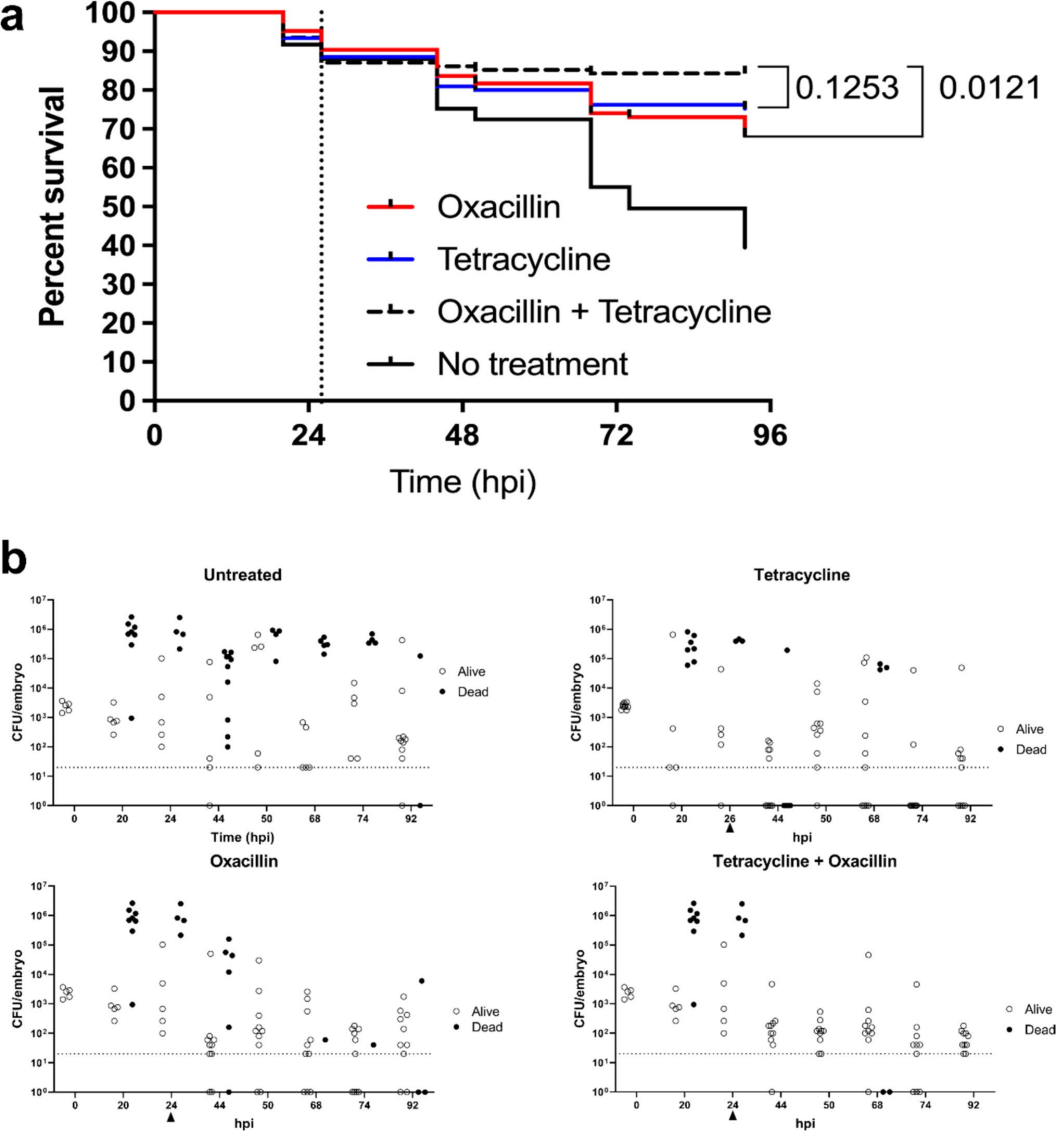
Combination antibiotic treatment increases survival of infected zebrafish larvae**. a** Kaplan–Meier survival curve of NewHG-infected London Wild-Type (LWT) zebrafish larvae treated at 1 dpi (dotted line) with 50 µg ml^-1^ tetracycline, 32 µg ml^-1^ oxacillin, both or left untreated. Treatment with both oxacillin and tetracycline significantly increases survival (*P* < 0.05, Log-Rank (Mantel-Cox) test) compared to embryos treated with oxacillin alone. n = 29-41 embryos per group. **b** Bacterial load in LWT zebrafish larvae untreated or treated at 1 dpi with 50 µg ml^-1^ tetracycline, 32 µg ml^-1^ oxacillin or both. Initial infectious doses were approximately 10^3^ CFU per embryo. Arrows indicate at what time treatment was added

We then used an established zebrafish larval model of *S. aureus* infection [20] to study the effects of this combination treatment in vivo. Treatments (50 µg ml^-1^ tetracycline and 32 µg ml^-1^ oxacillin) were given to NewHG *S. aureus* (MSSA)-infected larvae individually or together at 1 dpi, to determine if combination treatment could further rescue larvae compared to each treatment alone (Figure 3a). Previously, it had been demonstrated that these concentrations of tetracycline and oxacillin were curative or able to reduce bacterial load, respectively, in this model [5]. Combination treatment significantly (p = 0.0121) increased survival compared to oxacillin treatment. In this zebrafish model, the infection is controlled by the innate immune system or bacteria proliferate and kill larvae when numbers reach 10^6^ CFU per larva, at approximately 20 - 24 hpi [20, 21]. Bacterial numbers were determined in a sample of living larvae and all dead larvae at each timepoint to elucidate the growth dynamics of the bacteria in this model and understand if the treatments increased survival by controlling the growth of or killing bacteria, alongside the innate immune system (Figure 3b). Both individual treatments result in more tightly controlled infections, i.e. fewer larvae with bacterial numbers reaching a lethal threshold, and this effect was amplified when combination treatment was given, as the CFU per larva more clustered around 10^2^ – 10^3^ rather than a spread up to 10^6^ CFU (Figure 3b). With tetracycline treatment at 1 dpi, there were more dead larvae than the equivalent group treated at 6 hpi (Supplementary Figure S.2). *S. aureus* in 1 dpi oxacillin-treated larvae displays similar growth dynamics to 6 hpi treatment (Supplementary Figure S.2), suggesting that oxacillin can still treat more established infections including those where abscesses have begun to develop. We observe that oxacillin treatment results in a subpopulation of larvae where bacterial load is controlled around 10^2^ or 10^3^ CFU, which we postulate are intracellular persisters “hiding” in phagocytes [32, 33]. This demonstrates that the combination of tetracycline and oxacillin displays synergy in vitro against biofilms and increases antibiotic efficacy in vivo.

**Fig. 4:**
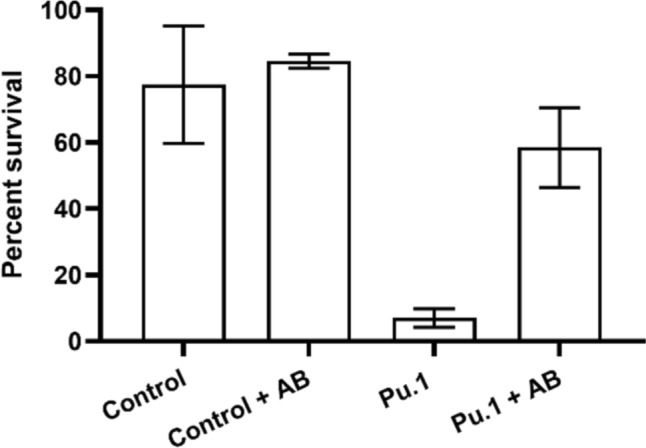
Tetracycline and oxacillin in combination rescue zebrafish larvae with knockdown phagocyte development**.** Percentage at 18 hpi of alive *Tg(BACmpx:gfp)i114* zebrafish larvae, injected at the 1-cell stage with control or pu.1 morpholino, infected with a target dose of 1500 CFU NewHG at 33 hpf and left untreated or immediately treated with 50 µg ml^-1^ tetracycline and 32 µg ml^-1^ oxacillin (AB). Although non-significant (Mann–Whitney test), addition of AB increased survival in the pu.1 morphants. Columns show mean of 2 biological repeats (n = 21-29 embryos per group), and error bars represent standard deviation

We next investigated the role of the innate immune system in conjunction with antibiotic combination treatment in vivo. In early stages of zebrafish development, the innate immune system is the sole defence against infection [22]. We tested whether the antibiotic combination controls the infection in conjunction with phagocytes to kill the bacteria, or if the antibiotics primarily control bacterial growth, with oxacillin being the primary bactericidal agent in the system. We injected pu.1 morpholino (pu.1 MO), a modified oligonucleotide, into single cell stage zebrafish larvae to knock down development of myeloid cells [34]. Without antibiotic treatment, survival of zebrafish larval morphants with knockdown phagocytes (pu.1 group) was significantly reduced compared to a standard control MO group (that had no disruption to innate immunity development [35]), due to uncontrolled bacterial proliferation [21] (Figure 4). This indicates that this treatment can partially mitigate the lack of phagocytes, and that in this scenario the antibiotics have more of an impact on controlling *S. aureus* infection than phagocytes.

## Conclusion

We have demonstrated that tetracycline and oxacillin display a synergistic relationship and are able to dramatically reduce the concentration of each antibiotic necessary to eradicate established *S. aureus* biofilms, and that this combination is highly efficient in a zebrafish model, including in phagocyte-depleted larvae. Growth arrest of *S. aureus* due to translation inhibition by tetracycline will result in decreased virulence determinant production, thereby decreasing its pathogenic potential in the zebrafish larvae. However, synergy between the two antibiotics in vitro indicates that this is unlikely to be responsible for our in vivo observations.

In the in vitro biofilm model, the concentration of each drug necessary to eradicate biofilm was reduced four times when the drugs were used in combination (Figure 2). Additionally, we demonstrated that there is a trend towards synergy in a variety of growth contexts (Supplementary Figure S.1). The fact that the combination was able to work so effectively against biofilms that were already grown shows potential for therapeutic use; with high demand for therapies that can treat implant-related infections and eradicate biofilm in patients [13], using existing antibiotics in a new way is one relatively simple approach to act on these problems. As both tetracyclines and oxacillin are routinely used to treat *S. aureus* infections, this is a combination that could be relatively simple to implement in clinical practice if synergy occurs in human patients, given their ease of access and relatively low toxicity [6–8]. As the two most commonly prescribed classes of antibiotics in the UK [8], if combining these drugs can additionally slow development of resistance in currently MSSA strains, this is another advantage.

In the zebrafish model, it was observed that oxacillin treatment alone resulted in a subpopulation of larvae that host a sublethal load of bacteria around 10^2^ or 10^3^, depending on treatment time (Figure 3b, Supplementary Figure S.3c). We hypothesise that this is due to the fact that oxacillin cannot enter phagocytes and is known to stimulate intracellular persister phenotypes [32, 33]. It is possible that when combined, oxacillin can kill the extracellular bacteria, and tetracycline can enter the phagocytes, preventing intracellular growth and allowing the innate immune system to exert greater control than without both drugs. Due to the fact that low concentrations of antibiotics can stimulate intracellular persisters, it is important to ensure that at least one of the antibiotics is able to enter phagocytes to access these bacteria and prevent intracellular growth [13]. When the phagocytes are knocked down with pu.1 MO, there are no intracellular bacteria, and the drugs can work on the same population of bacteria at the same time. It has been established that combining β-lactam antibiotics with aminoglycosides, which like tetracycline target the ribosome, is synergistic as the effects on the cell wall by the β-lactams allow the aminoglycosides to enter the cell more freely [2]. It is likely that such an effect could be occurring with the tetracycline and oxacillin combination but additional further work is required to understand the mechanism behind the synergy observed. Furthermore, extension of our combinatorial approach in the zebrafish model may identify other, clinically relevant, antibiotic pairs that demonstrate synergy, dependent on the toxicity of the compounds and their ability to penetrate the fish.

Future work would involve expanding upon the in vivo model to determine by how much the curative doses could be decreased when tetracycline and oxacillin are used in combination and investigating the efficacy of this combination in a mammalian model (for example, murine) and ultimately to determine whether this effect is seen in human clinical trials. Additionally, it would be pertinent to establish whether combining β-lactam antibiotics with tetracycline could re-sensitise MRSA strains to such drugs they are otherwise resistant to. Finally, as one goal of combining antibiotic treatments is to slow development of resistance to the drugs individually, further work is required to determine if or how quickly resistance develops in MSSA strains such as UAMS-1 and NewHG to the oxacillin–tetracycline combination.

## Supplementary Information

Below is the link to the electronic supplementary material.

Supplementary file1 (DOCX 13 KB)

Supplementary file2 (DOCX 13 KB)

## Data Availability

The datasets generated during and/or analysed during the current study are available from the corresponding author on reasonable request.
