# Supplementary material for: Tetracycline and Oxacillin Act Synergistically on Biofilms and Display Increased Efficacy In Vivo Against Staphylococcus aureus
Source: Curr Microbiol. 2024 Nov 6;81(12):447. doi: 10.1007/s00284-024-03959-4 (PMC11541413; doi:10.1007/s00284-024-03959-4)
Supplement: Supplementary file 1 — Supplementary file1 (DOCX 13 KB) [file 284_2024_3959_MOESM1_ESM.docx]

**SFig. S.1** Oxacillin and tetracycline display synergy *in vitro***. a** E test for synergy. E test strips were applied to Mueller Hinton agar (MHA) plates seeded with *S. aureus* NewHG to find the MIC of tetracycline (1 µg ml^-1^) and oxacillin (0.19 µg ml^-1^). This was repeated in combination crossing at the MICs, resulting in decreased MICs for both antibiotics (0.125 µg ml^-1^ and 0.25-0.5 µg ml^-1^ respectively), indicating synergy. **b** Disk diffusion assay for synergy. MHA plates were prepared as in A. 1 µg oxacillin and 30 µg tetracycline were applied to filter paper disk and placed on MHA plates seeded with *S. aureus* NewHG. Top left: oxacillin and tetracycline control: the discs are placed too far apart for the antibiotics to interact. Top right: interaction between oxacillin and tetracycline indicates a synergistic relationship. Bottom row: controls with oxacillin/oxacillin and tetracycline/tetracycline placed at the same distance as the top right plate. **c** Checkerboard assay with oxacillin and tetracycline: In this assay, individual MIC values were 0.08 µg ml^-1^ for oxacillin and 0.125 µg ml^-1^ for tetracycline (highlighted in yellow). Sub MIC conditions are highlighted in red. In combination these concentrations reduced to 0.005 µg ml^-1^ and 0.0625 µg ml^-1^ respectively (outlined in red). These values were used to calculate the FIC index which in this case was 0.5625. The FIC value is defined as additive or indifferent, but close to the values that would be classified as synergistic

**SFig. S.2** Oxacillin and tetracycline combination treatment at 6 hpi decreases bacterial load in zebrafish**.** NewHG infected LWT zebrafish embryos were **a** untreated, **b** treated with 50 µg ml^-1^ tetracycline at 6 hpi, **c** treated with 32 µg ml^-1^oxacillin at 6 hpi, or **d** treated with both 32 µg ml^-1^ oxacillin and 50 µg ml^-1^ tetracycline at 6 hpi and survival monitored over 92 hours. Initial infectious doses were approximately 10^3^ CFU per embryo. CFU per embryo was determined by sampling 5-10 living embryos and every dead embryo per timepoint, homogenising embryos and plating serial dilutions onto TSA, growing at 37°C and counting the colonies
