# Supplementary material for: Tetracycline and Oxacillin Act Synergistically on Biofilms and Display Increased Efficacy In Vivo Against Staphylococcus aureus
Source: Curr Microbiol. 2024 Nov 6;81(12):447. doi: 10.1007/s00284-024-03959-4 (PMC11541413; doi:10.1007/s00284-024-03959-4)
Supplement: Supplementary file 2 — Supplementary file2 (DOCX 13 KB) [file 284_2024_3959_MOESM2_ESM.docx]

**E-test**

4-5 fresh colonies of *S. aureus* NewHG that had been grown overnight at 37°C on TSA were added to 2 ml PBS to obtain a cloudy suspension. MHA (Mueller-Hinton agar) plates were swabbed with the suspension of *S. aureus* NewHG. E-test strips (bioMérieux) were applied to the surface of the agar and the plates were incubated at 37°C overnight. The MIC of each individual antibiotic was determined as indicated on the strip at the lowest concentration where growth was inhibited. Fresh plates were prepared as before and the E-test strips were overlapped at 90° at their individual MICs and incubated overnight at 37°C. The following morning the MICs in combination with each other were read off the strips.

**Disk diffusion assays**

MHA plates were seeded with the bacterial suspension with a sterile swab as for the E test.

Filter paper disks that had 10 µl of antibiotics of standard concentration (100 µg ml^-1^ oxacillin and 3000 µg ml^-1^ tetracycline) were applied to each plate so that the antibiotic would diffuse into the agar. Plates were incubated overnight at 37°C. Growth was prevented at inhibitory concentrations of the antibiotic in the agar. To determine if synergy existed between two antibiotics, the filter paper disks were applied 20 mm apart so that their zones of inhibition would touch at the edges. When synergy occurred the two circles overlapped with an expanded zone of inhibition.

**Checkerboard assay**

A 96-well plate (plate 1) was set up containing 1.1 µg ml^-1^ oxacillin in column 1 (200 µl each well). 100 µl TSB was added to columns 2-9, and serial 2 fold dilutions were carried out from columns 1 to 9, and 100 µl TSB was added to column 10 (no antibiotics). A second plate was set up with a gradient of tetracycline (4.4 µg ml^-1^ in row A) moving down the rows A to G with row H containing no antibiotic. 45 µl from each well of both plates was transferred to the corresponding well of a third plate to give 90 µl per well. 90 µl TSB was added to column 11 and 100 µl TSB was added to column 12. 10 µl of bacterial overnight culture diluted to OD_600_ 0.01 was added to every well except row 12. Row 11 was therefore a positive control for uninhibited bacterial growth and row 12 contained blank TSB. The plate was sealed with parafilm and put in a plastic box containing a layer of paper towel soaked in PBS to prevent the plate from drying out. The plastic box was sealed with parafilm (Bemis). The plate was incubated at 37°C for 24 hours and the OD_600_ was measured using column 12 as a blank using a Hidex Sense microplate reader (Hidex Oy).
